# LincSNP: a database of linking disease-associated SNPs to human large intergenic non-coding RNAs

**DOI:** 10.1186/1471-2105-15-152

**Published:** 2014-05-20

**Authors:** Shangwei Ning, Zuxianglan Zhao, Jingrun Ye, Peng Wang, Hui Zhi, Ronghong Li, Tingting Wang, Xia Li

**Affiliations:** 1 College of Bioinformatics Science and Technology, Harbin Medical University, Harbin 150081, China

**Keywords:** LincRNA, Disease-associated SNPs, GWAS, Non-coding RNA, Database

## Abstract

**Background:**

Genome-wide association studies (GWAS) have successfully identified a large number of single nucleotide polymorphisms (SNPs) that are associated with a wide range of human diseases. However, many of these disease-associated SNPs are located in non-coding regions and have remained largely unexplained. Recent findings indicate that disease-associated SNPs in human large intergenic non-coding RNA (lincRNA) may lead to susceptibility to diseases through their effects on lincRNA expression. There is, therefore, a need to specifically record these SNPs and annotate them as potential candidates for disease.

**Description:**

We have built LincSNP, an integrated database, to identify and annotate disease-associated SNPs in human lincRNAs. The current release of LincSNP contains approximately 140,000 disease-associated SNPs (or linkage disequilibrium SNPs), which can be mapped to around 5,000 human lincRNAs, together with their comprehensive functional annotations. The database also contains annotated, experimentally supported SNP-lincRNA-disease associations and disease-associated lincRNAs. It provides flexible search options for data extraction and searches can be performed by disease/phenotype name, SNP ID, lincRNA name and chromosome region. In addition, we provide users with a link to download all the data from LincSNP and have developed a web interface for the submission of novel identified SNP-lincRNA-disease associations.

**Conclusions:**

The LincSNP database aims to integrate disease-associated SNPs and human lincRNAs, which will be an important resource for the investigation of the functions and mechanisms of lincRNAs in human disease. The database is available at http://bioinfo.hrbmu.edu.cn/LincSNP.

## Background

Identification of genetic variants that underlie complex traits is one of the main tasks of current genetic research [1]. In recent years, genome-wide association studies (GWAS) have identified thousands of genetic variants that are associated with a wide spectrum of diseases (or phenotypes). However, many single nucleotide polymorphisms (SNPs), the most common type of genetic variant, are located in the intergenic regions, which makes it difficult to clarify their functions and involvement in human disease [2]. Recently, a small number of studies have begun to create a relationship between disease-associated SNPs and large intergenic non-coding RNAs (lincRNAs) [3]. These lincRNAs are greater than 200 nucleotides in length and have been shown to play a critical role in many key biological processes [4,5]. The number of human lincRNAs continues to increase and many studies have demonstrated a role in a wide variety of diseases, such as cancer [6,7].

In previous studies, disease-associated SNPs in microRNAs and microRNA target sites have been widely investigated [8,9]. MicroRNAs (miRNAs) represent an abundant class of small non-coding RNAs that regulate gene expression by binding mostly to the 3′-untranslated region of mRNA. This results in RNA degradation or translational repression [10]. The role of miRNA polymorphisms in human diseases has been well established in both experimental and bioinformatic analyses [11,12]. Recently, the linkage of disease-associated SNPs with human lincRNAs has become a new area of interest. For example, a recent study of papillary thyroid carcinoma (PTC) found that a PTC-associated SNP, rs944289, identified by GWAS, is located 3.2 kb upstream of a lincRNA (*PTCSC3*). This SNP could affect the expression of *PTCSC3*, which indicates a potential mechanism behind susceptibility to PTC [13]. Another study performed a meta-analysis of two existing results from GWAS. It identified a new SNP, rs3787016, which is associated with prostate cancer and is found in a lincRNA region [14]. A different study found a SNP, rs7763881, in *HULC* lincRNA, which could contribute to decreased susceptibility to hepatocellular carcinoma in HBV persistent carriers [15]. In addition, GWAS have identified several disease-associated SNPs in a lincRNA called *ANRIL*[3] and have provided more examples where SNPs affect lincRNA expression [16]. This growing list of related studies will provide a clearer blueprint of the extent and precise mechanism of lincRNA polymorphisms in various human diseases. Therefore, it is necessary to identify and understand these potential functional variants that reside in human lincRNAs.

**Figure 1:**
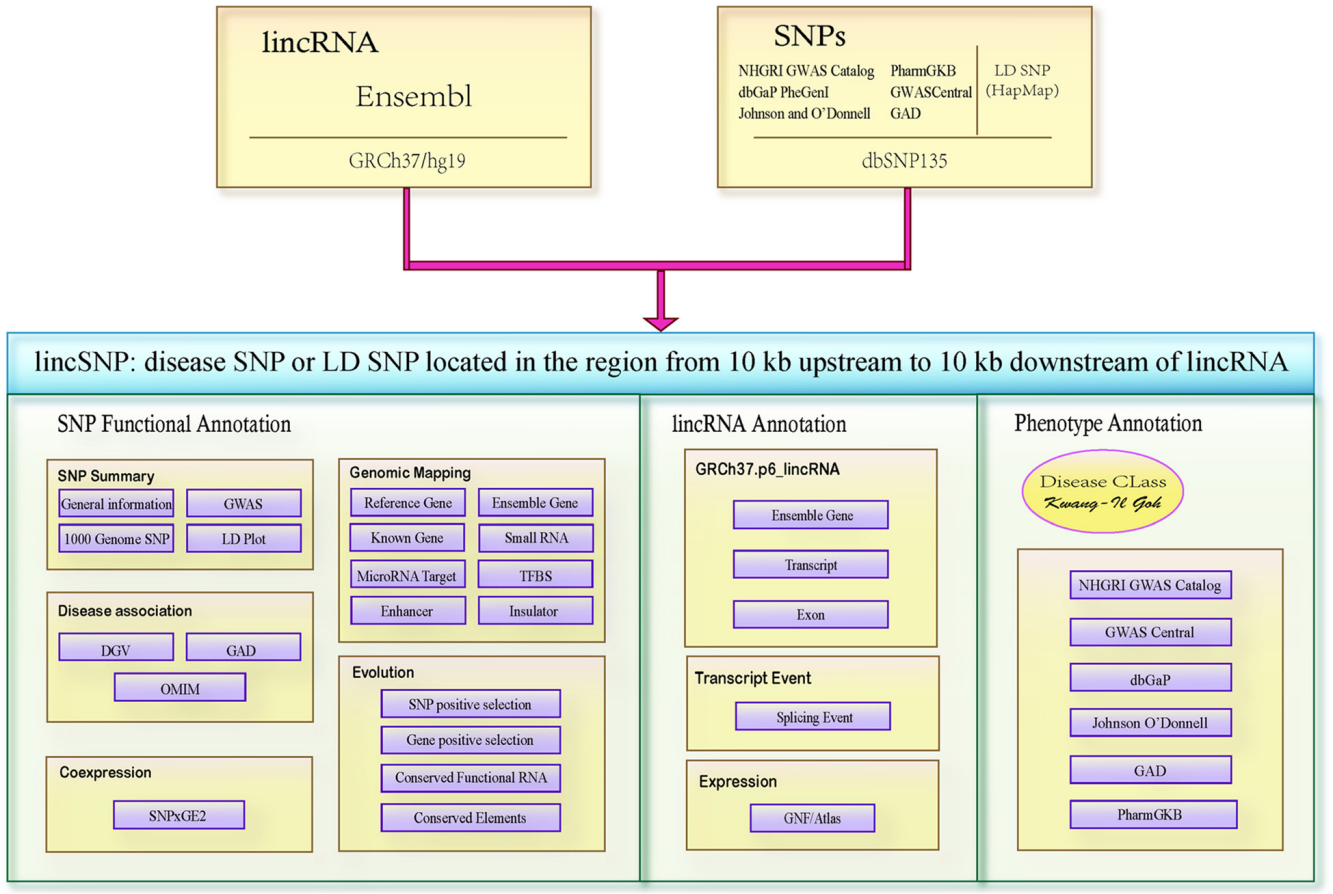
**Overall structure of the LincSNP database.** LincSNP integrates the annotation of three groups: disease-associated SNPs, lincRNAs and diseases.

In our previous work, we have performed an analysis of lincRNA polymorphisms and identified disease-associated SNPs in these regions [17]. Therefore, we anticipate that lincRNAs can be mapped to more disease-associated SNPs. In this study, we developed a practical and user-friendly database called LincSNP (available at http://bioinfo.hrbmu.edu.cn/LincSNP), which is a comprehensive data source for integrating current lincRNA and GWAS SNP annotations. The overall structure of LincSNP is shown in Figure 1. Linkage disequilibrium (LD) analysis has been used and greater than 1.5 million annotated SNPs are stored in the current version of LincSNP. Approximately 140,000 SNPs can be associated with approximately 5,000 human lincRNAs. The database makes an attempt to bridge the gap between disease-associated SNPs and human lincRNAs. This will enhance our understanding of lincRNA function, particularly the potential role of lincRNAs in human disease.

## Construction and content

### Data sources

We downloaded approximately 5,700 human lincRNAs from the Ensembl database, together with their annotations (Ensembl version 68, Homo sapiens assembly GRCh37.p8), such as gene feature, transcripts, exons, transcription events and expression. Disease (phenotype) associated SNPs were integrated using six data sources: dbGAP [18], GAD [19], GWAS Central [20], Johnson and O’Donnell [21], NHGRI GWAS Catalog [22] and PharmGKb [23] (versions shown in Table 1). These data sources have strict criteria to filter published GWAS studies and ensure the dependability of the data for further analysis. Previous studies have shown that many true disease-associated SNPs do not have stringent *P*-values [24]. Thus, to increase the number, we selected disease-associated SNPs from original publications with moderate thresholds (*P*-values < 1.0 × 10^−3^). If data for the same SNP could be obtained from different publications, we selected only the most significant data set [25]. In addition, we manually collected SNP-lincRNA-disease associations from publications taken from the PubMed database (published before April 1st, 2014), where experimental evidence was given. Experimentally supported lincRNA-disease associations from a previous study were also collected and annotated in the LincSNP database [26].

**Table 1:** Version of GWAS data sources

**Source**	**Link**	**Version**
dbGAP	http://www.ncbi.nlm.nih.gov/gap	Aug/2012
GAD	http://geneticassociationdb.nih.gov/cgi-bin/index.cgi	Jun/2013
GWAS Central	http://www.gwascentral.org	Mar/2013
Johnson and O’Donnell [21]	http://www.ncbi.nlm.nih.gov/pubmed/19161620	Jan/2009
NHGRI GWAS Catalog	http://www.genome.gov/gwastudies	Aug/2011
PharmGKb	http://www.pharmgkb.org/index.jsp	Apr/2013

In total, 128,407 unique disease-associated SNPs were collected. We also extracted SNPs that had linkage disequilibrium (LD SNP, r^2^ ≥ 0.5 in at least one population) relationships with disease-associated SNPs from the HapMap CEU, HCB + JPT and YRI populations (release #28). After LD analysis, approximately 1.5 million potential disease-associated SNPs or LD SNPs was collected in LincSNP. We performed comprehensive annotation for these SNPs using currently available annotation information. A detailed list that describes the annotation sources is provided in Table 2.

**Table 2:** Sources of disease-associated SNP annotation

**Annotation**	**Item**	**Source**	**Description**
SNP Summary	General Information	BioQ	BioQ provides query and documentation tools for genomic relational databases.
	Genome Wide Association	BioQ	
	1000 Genome SNP	BioQ	
	LD Plot	BioQ	
Genomic Mapping	Reference Gene	UCSC	Genes and Gene Prediction Tracks\RefSeq Genes\refGene;
	Ensemble Gene	UCSC	Genes and Gene Prediction Tracks\Ensembl Genes\ensGene;
	Known Gene	UCSC	Genes and Gene Prediction Tracks\UCSC Genes\knownGene;
	Small RNA	UCSC	Genes and Gene Prediction Tracks\sno/miRNA\wgRna;
	MicroRNA Target	UCSC	Regulation\TS miRNA sites\targetScanS;
	TFBS	UCSC	Regulation\TFBS Conserved\tfbsConsSites;
	Enhancer	VISTA Enhancer	Tissue specific human enhancers;
	Insulator	CTCFBSDB	Vertebrate genomic insulators;
Evolution	SNP and Gene positive selection	SNP@Evolution	A hierarchical database of positive selection on the human genome;
	Conserved Functional RNA	UCSC	Genes and Gene Prediction Tracks\EvoFold\evofold;
	Conserved Elements	UCSC	Comparative Genomics\Conservation\Mammal EI;
Gene Co-Expression	3-way SNP-expression Associations	SNPxGE2	Human SNP–expression associations
Disease Association	OMIM gene	OMIM	Online Mendelian Inheritance in Man;
	DGV gene	DGV	Structural variation in the human genome;
	GAD gene	GAD	Genetic Association Database;

**Note:** BioQ: http://bioq.saclab.net; UCSC: http://genome.ucsc.edu; VISTA Enhancer: http://enhancer.lbl.gov; CTCFBSDB: http://insulatordb.uthsc.edu; SNP@Evolution: http://bighapmap.big.ac.cn; SNPxGE2: http://lambchop.ads.uga.edu/snpxge2/index.php; OMIM: http://www.ncbi.nlm.nih.gov/omim; DGV: http://dgv.tcag.ca/dgv/app/home; GAD: http://geneticassociationdb.nih.gov/cgi-bin/index.cgi.

### Mapping disease-associated SNPs to human lincRNAs

One of the main features of LincSNP is that all disease-associated SNPs from existing studies that can be mapped to human lincRNAs are identified and annotated. It has also been found that disease-associated SNPs in the up- and down-stream regions of human lincRNA may be potential functional variants. They may disrupt some functional elements, such as transcription factor binding sites (TFBSs), and lead to disease by changing the expression of lincRNA [13]. Thus, we identified all disease-associated SNPs located in human lincRNA regions and within 10 kb up- and down-stream of lincRNAs [27]. Of the 128,407 SNPs associated with various diseases or phenotypes, 11,631 were mapped to 3,323 human lincRNAs. After LD analysis, a total of 128,785 LD SNPs (r^2^ ≥ 0.5) were mapped to 4,906 human lincRNAs.

### Database construction

The LincSNP database is composed of a web interface and a MySQL database management system. The MySQL (version 5.1) system was used to store and manage all data in LincSNP. The data processing programs were written in Java (version 1.6.0), the web interfaces were built in JSP and jQuery plugins were used for the interface development. The web services were developed using Apache Struts2 (version 2.1.8), which is a Java web application framework.

We built user-friendly web interfaces that allow users to perform free text searches and download data sets in the LincSNP database. The searchable terms include disease/phenotype name, lincRNA name (Ensembl ID), SNP ID (rs) and chromosome region. We provide alternative query options (disease-associated SNPs located in the lincRNAs or different distances from lincRNAs) so that users have the freedom to query disease-associated SNPs in the lincRNAs or up- and down-steam of lincRNAs. To collect new, experimentally supported SNP-lincRNA-disease associations, we also provide a web interface for users to submit novel data into the database. Search results are returned as a list of SNP IDs (rs) that correspond to a ‘Result information’ page, which can be optionally displayed in a number of sections based on selected fields. These sections include: an ‘SNP’ section for basal information on the inquired SNP and other annotated LD SNPs related to this initial SNP; a ‘lincRNA’ section that contains the annotations for the lincRNAs; and a ‘Phenotype’ section that contains the GWAS information, such as phenotype name, original *P*-values and references. Details of these sections and subsections are described in the Help page of the LincSNP database.

## Utility and discussion

Discovery of the roles of genetic variants in common diseases is currently the subject of intense research. The most common variants are SNPs, which have been used as biomarkers for disease association and susceptibility. Within coding regions, SNPs can affect protein functions directly, by changing the amino acid sequences or by disrupting their regulation. When SNPs are located in non-coding RNA sequence, they may be involved in different mechanisms. Currently, lincRNAs have been identified as a class of non-coding functional transcripts and it is becoming increasingly clear that disease-associated SNPs can reside in these regions. Several previous studies have integrated GWAS and miRNA data and have provided resources for miRNA related SNP studies [11,28]. Therefore, we developed the LincSNP database to provide researchers with a time- and cost-efficient bioinformatic tool to query candidate disease-associated SNPs in human lincRNAs.

**Figure 2:**
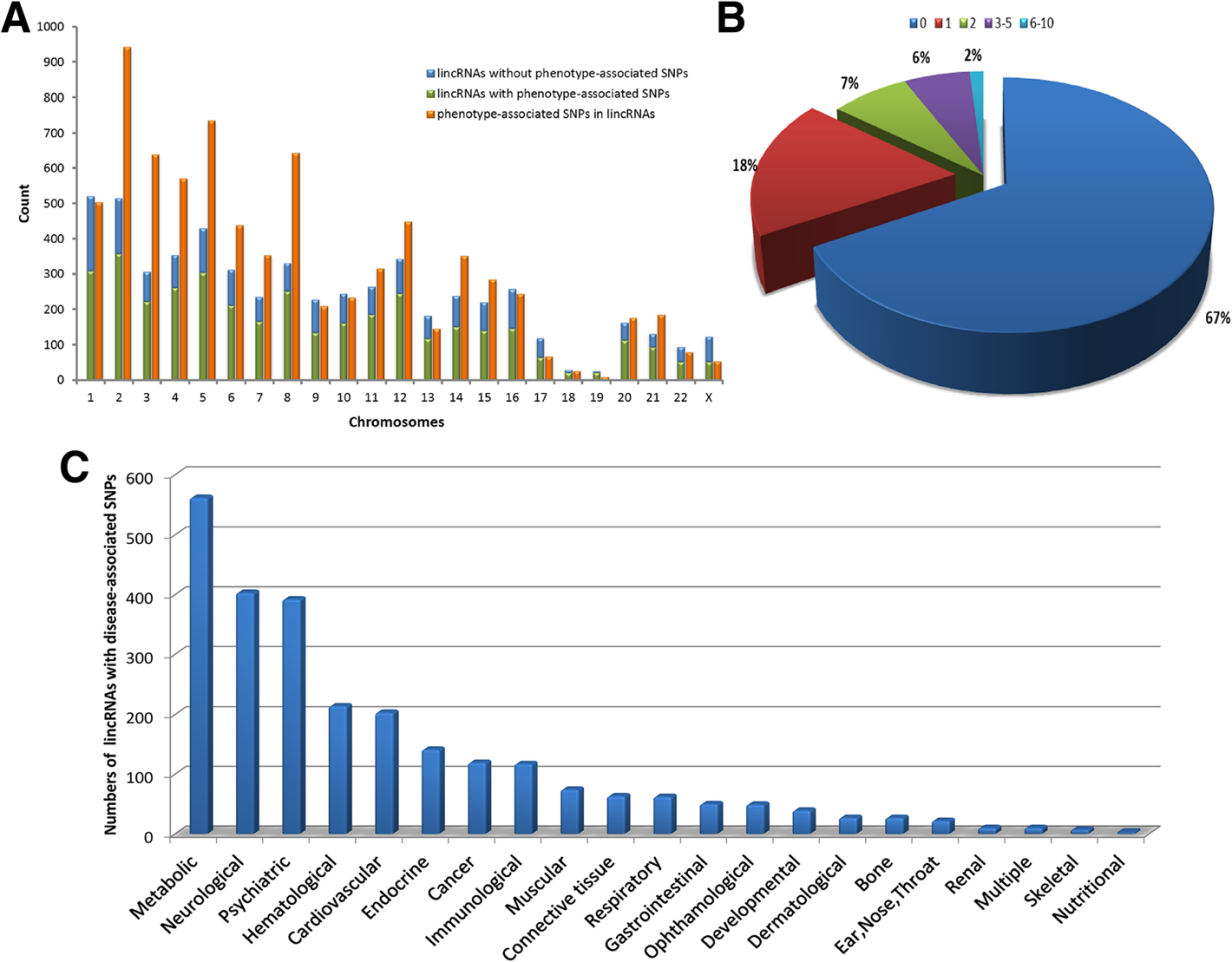
**Distribution of disease-associated SNPs in human lincRNAs. (A)** The distribution of lincRNAs and disease (phenotype) associated SNPs, classified by chromosomes. Green bars represent the lincRNAs with disease (phenotype) associated SNPs, blue bars represent other lincRNAs and orange bars represent disease-associated SNPs in human lincRNAs. **(B)** The distribution of lincRNAs with different numbers of disease-associated SNPs. **(C)** The distribution of disease-associated SNPs in human lincRNAs. These diseases (phenotypes) were classified into 21 classes, in accordance with the criterion published by Goh et al.

Using data from LincSNP, we found that many human lincRNA regions could be mapped to disease-associated SNPs (Figure 2A). Approximately one third of human lincRNAs were mapped to at least one disease-associated SNP and several lincRNAs were mapped to more than six disease-associated SNPs (Figure 2B). In addition, we identified all disease (phenotype) associated SNPs that could be mapped to human lincRNAs and classified the diseases into 21 different classes (unclassified not shown), using the classification scheme from a previous study [29]. We then investigated whether human lincRNAs were more likely to take part in specific disease classes. We found that metabolic, neurological and psychiatric diseases were the top three potential lincRNA related classes (Figure 2C).

**Figure 3:**
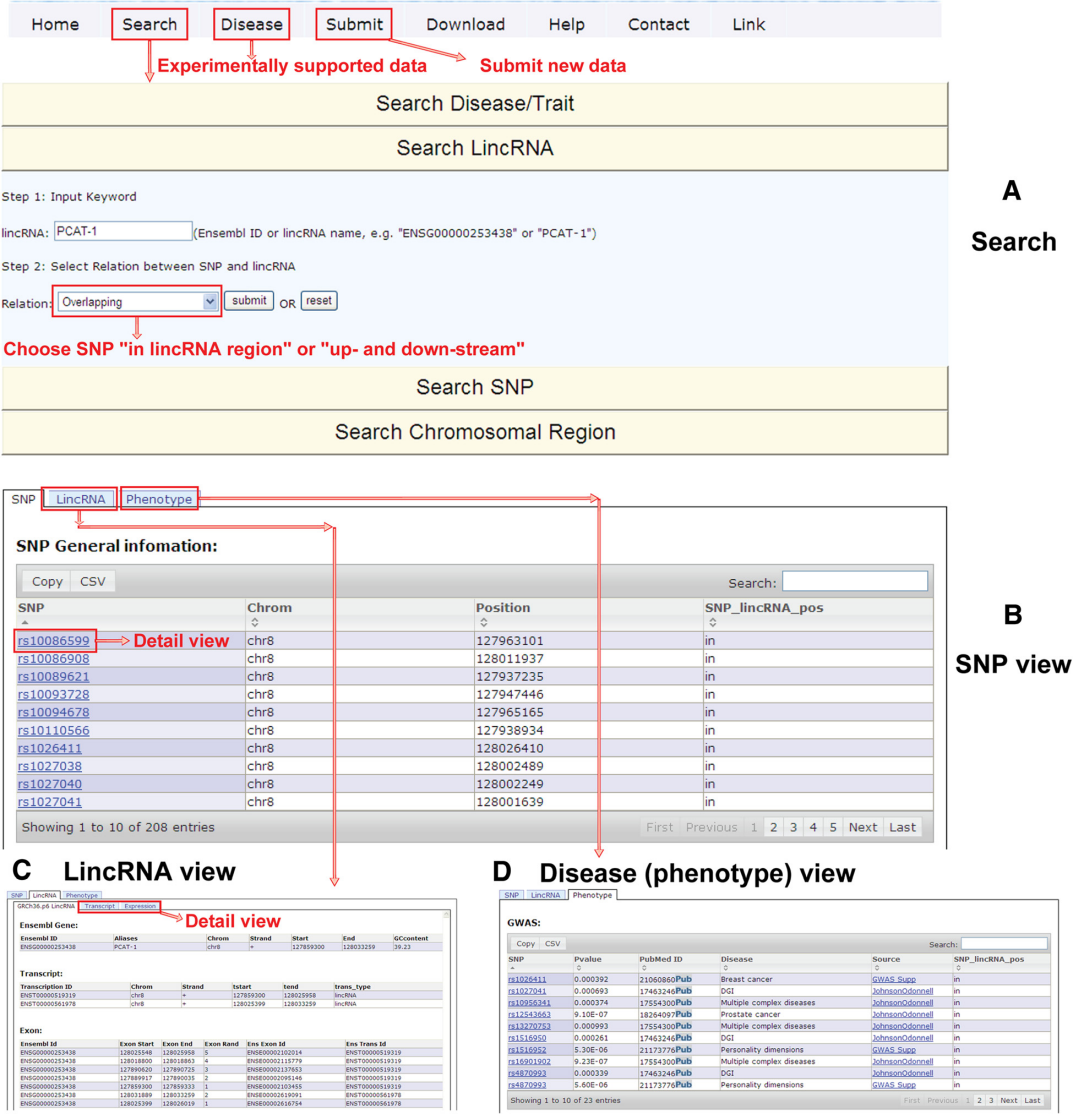
**Overview of the LincSNP web interface. (A)** An example: screenshot of the main search page, search for lincRNA *PCAT-1* (*ENSG00000253438*). The corresponding result pages are shown. **(B)** A list of SNPs in or around *ENSG00000253438* is shown in the ‘SNP’ section, **(C)** the ‘lincRNA’ section contains three subsections: ‘GRCh36.p6_lincrna’, ‘Transcript’ and ‘Expression’, and **(D)** the ‘phenotype’ section provides information on the disease-associated SNPs and sources.

One of the potential applications of LincSNP is to predict new disease-associated lincRNAs, based on SNPs that are already known to be associated with certain diseases. For example, a previous GWAS study has identified an SNP (rs12543663) associated with prostate cancer susceptibility [30]. Using the LincSNP database, we found that this SNP is located in a newly annotated lincRNA, *PCAT-1* (*ENSG00000253438*). This lincRNA has recently been demonstrated to be a transcriptional repressor implicated in a subset of prostate cancer patients (Figure 3) [31]. Another application of LincSNP is to find the principles behind specific lincRNAs and diseases. For example, we found that human lincRNAs can be divided into three categories based on our genome-wide disease-associated SNP mapping. Firstly, several lincRNAs, such as *ENSG00000256166* and *ENSG00000214894*, were found to have multiple SNPs that were associated with specific diseases or phenotypes. These two lincRNAs were mapped to many disease-associated SNPs, most of which are associated with immunological disease. Secondly, some lincRNAs are enriched for disease-associated SNPs that are linked to multiple classes of disease. Examples of this are *ENSG00000232080*, *ENSG00000237838* and *ENSG00000242996*, which were mapped to SNPs associated with metabolic, endocrine, immunological, neurological and other diseases. Lastly, many lincRNAs were not mapped to any disease-associated SNPs, which suggested that SNPs in these lincRNAs were either lethal mutations or had no effect on diseases or phenotypes. These findings will provide novel insight into the roles of lincRNAs in human disease.

## Conclusions

LincSNP is designed as a comprehensive resource for linking disease-associated SNPs to human lincRNAs. We manually collected experimentally supported SNP-lincRNA-disease associations in the LincSNP database. Although the current number is limited, with the growth of interest in human lincRNAs and the availability of high-throughput technologies, the total number of disease-associated lincRNAs and SNPs will undoubtedly continue to grow. We will recruit new disease-associated lincRNAs and SNPs and update the LincSNP database in a timely manner. We will also incorporate new functional annotations and more data sources to improve the utility of this database. These strategies will make the data more comprehensive and improve the performance of LincSNP, to make it increasingly useful for future studies.

## Availability and requirements

LincSNP is freely available on the web at http://bioinfo.hrbmu.edu.cn/LincSNP.

## Abbreviations

GWAS: Genome-wide association studies; SNP: Single nucleotide polymorphism; lincRNA: Large intergenic non-coding RNA; LD: Linkage disequilibrium; CEU: Samples of Utah residents with Northern and Western European ancestry from the CEPH collection; HCB: Samples of Han Chinese in Beijing; JPT: Samples of Japanese in Tokyo; YRI: Samples of Yoruba people in Ibadan.

## Competing interests

The authors declare that they have no competing interests.

## Authors’ contributions

XL conceived of the project. SN, ZZ, HZ, RL and TW participated in the collection and analysis of all data sources. JY and PW designed and implemented the database. SN, ZZ and XL wrote the manuscript. All authors have read and approved the final manuscript.
